# Optimal Visualization of Os Subfibulare Using 3D Water Selective Cartilage Scan (3D_WATSc) MRI Sequencing: A Case Report

**DOI:** 10.7759/cureus.27469

**Published:** 2022-07-29

**Authors:** Aaron Cedric D Llanes, Dane Van Tassel, Alexxa Wirth, Luis F Goncalves, Mohan V Belthur

**Affiliations:** 1 Orthopaedic Surgery, University of Arizona College of Medicine-Phoenix, Phoenix, USA; 2 Radiology, Phoenix Children's Hospital, Phoenix, USA

**Keywords:** ankle, accessory ossicle, broström procedure, mri, os subfibulare

## Abstract

Os subfibulare is an accessory ossicle of the lateral malleolus at the distal end of the fibula. In most instances, os subfibulare is found incidentally on radiographs. While os subfibulare typically remains asymptomatic, some cases may present with ankle pain or instability. To initiate appropriate treatment and maximize patient outcomes, it is crucial to accurately visualize the accessory ossicle. Here, we report a symptomatic case of os subfibulare diagnosed with ankle radiographs and a 3D water selective cartilage scan (3D_WATSc, Ingenia, Philips Healthcare, The Netherlands) magnetic resonance imaging sequence and treated surgically with open ossicle excision and a modified Broström procedure.

## Introduction

Os subfibulare is a supernumerary bone of the lateral malleolus at the distal end of the fibula found in 1% of the general human population, usually in adolescents [1]. Difficulties in diagnosing os subfibulare result from difficulties in establishing the appropriate etiology, especially in children and adolescents [2]. In most instances, os subfibulare is found incidentally on ankle radiographs, but it is difficult to distinguish between an avulsion fracture and a secondary ossification center at the tip of the lateral malleolus that has not fused. While it typically remains asymptomatic, some cases of os subfibulare may present with ankle pain or instability, usually due to overuse or acute trauma to the ankle [3]. These symptoms are believed to be caused by impingement of the ossicle on surrounding tissue and irritation of the adjacent synovium and/or ligaments or from activity-related movement at the synchondrosis between the lateral malleolus and the ossicle [4].

Intervention for os subfibulare can range from conservative treatment, consisting of orthoses and/or physical therapy, to operative treatment. When conservative treatment fails to resolve the initial symptoms, surgery is indicated to prevent or address chronic pain and ankle instability. Depending on the size of the ossicle when excised, a Broström procedure, an anatomical reconstruction of the lateral ankle ligaments, particularly the anterior talofibular ligament (ATFL), may be necessary [5]. To initiate appropriate treatment of os subfibulare and maximize patient outcomes, it is crucial to accurately visualize the accessory ossicle and also understand the reason for persisting symptoms. We report a symptomatic case of os subfibulare diagnosed using radiographic imaging and 3D water selective cartilage scan (3D_WATSc, Ingenia, Philips Healthcare, The Netherlands) magnetic resonance imaging (MRI) sequencing and treated surgically with open ossicle excision and a modified Broström procedure.

## Case presentation

A 12-year-old female presented with pain and swelling in her left ankle. Physical examination of the lower extremity revealed slight swelling over the left distal fibular styloid process with lateral ankle instability and increased inversion on the left side.

**Figure 1:**
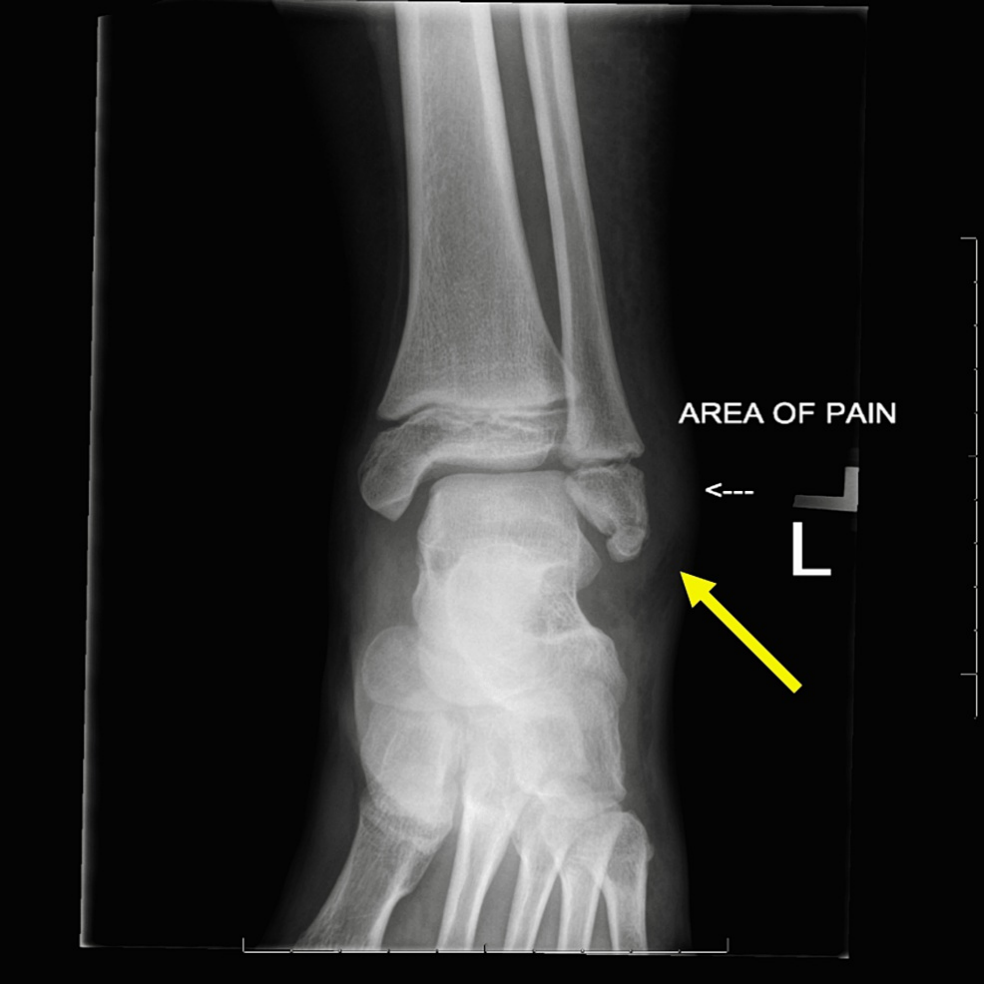
Anterior-posterior (AP) radiograph of the left ankle shows a fracture line and a reported area of pain at the distal lateral malleolus (yellow arrow) two years prior to presentation.

Two years prior, she presented with left ankle pain after playing on a trampoline. Ankle radiographs revealed an artifact at the left distal fibular epiphysis (Figure 1). This was diagnosed as a minimally displaced subacute fracture and was treated conservatively with a controlled ankle movement (CAM) walking boot. Follow-up imaging four weeks after the initial injury suggested the persistence of a fracture line, with slight increases in sclerosis suggesting healing of the fracture. The patient also reported improvement in pain at the time.

**Figure 2:**
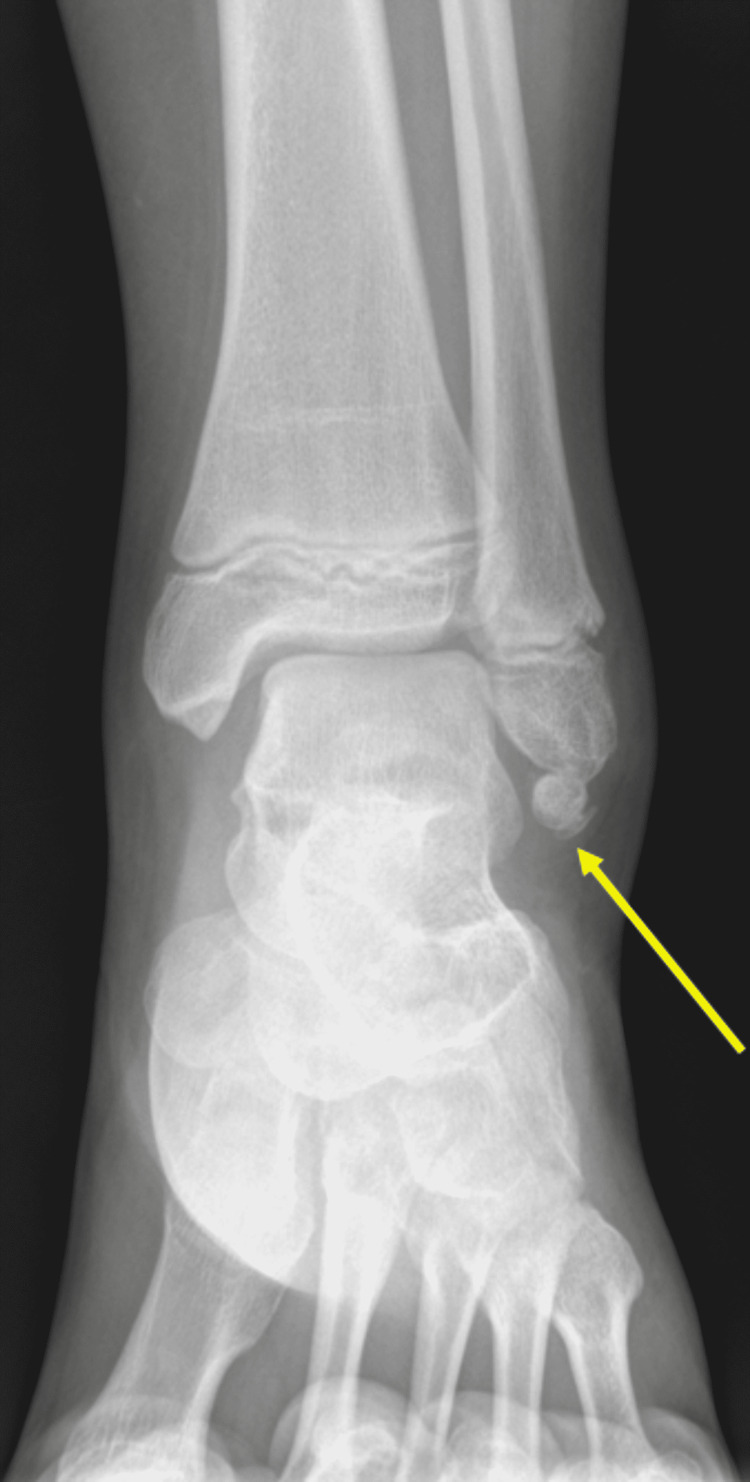
AP radiograph of the left ankle shows ossified fragments at the distal lateral malleolus (yellow arrow), similar to the appearance on prior radiographs. AP: anterior-posterior.

The patient returned to the clinic two years after the initial injury because the ankle pain and instability did not resolve with conservative management. Radiographs at this time demonstrated ossific fragments at the distal tip of the fibula comparable to the exam from two years prior, and the question of variant ossicles was posed (Figure 2).

**Figure 3:**
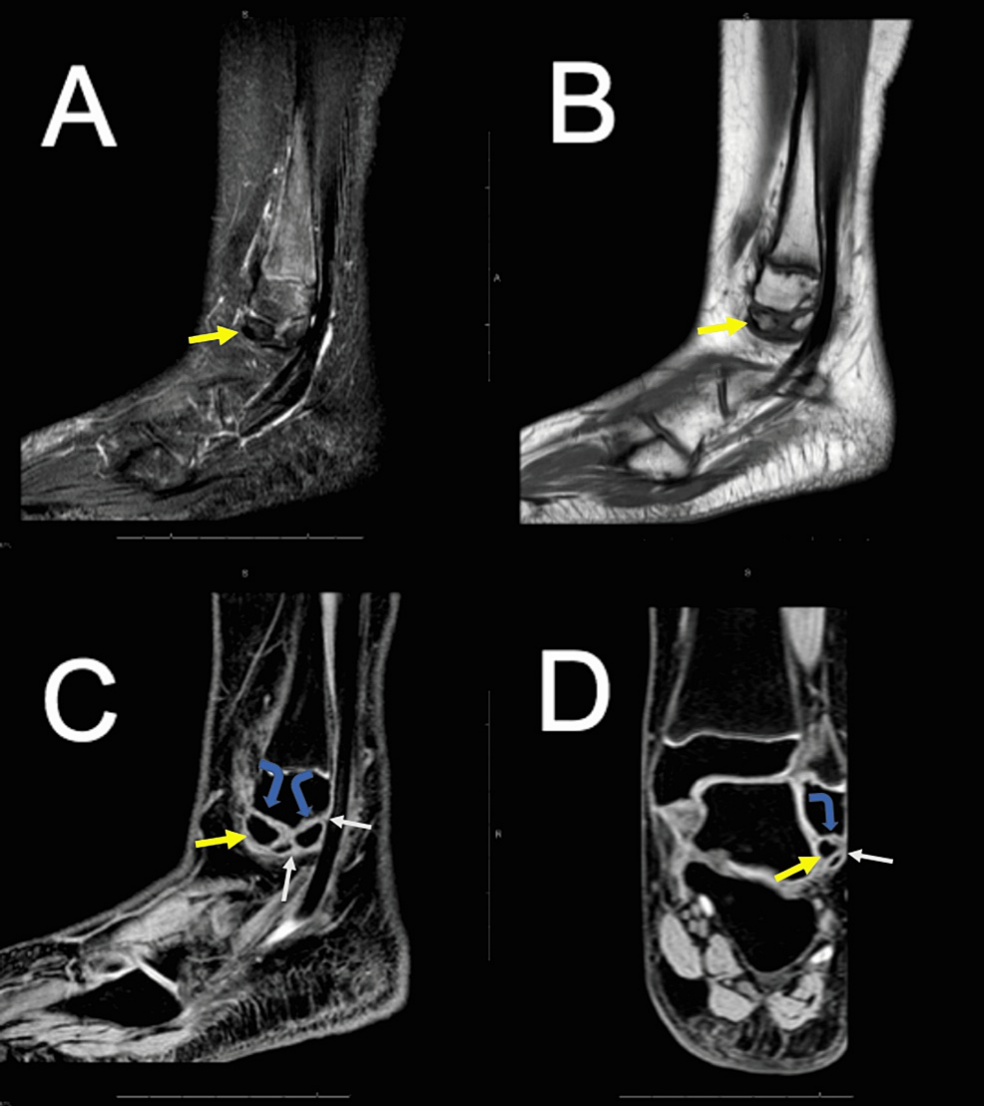
Sagittal T2 fat sat (A), sagittal T1 without fat sat (B), sagittal 3D_WATSc (C), coronal 3D_WATSc (D) sequences of the left ankle through the distal fibula demonstrating accessory ossicles centered along the distal tip of the fibula (yellow arrows). WATSc sequences best illustrate the cartilage signal surrounding the ossicles (white arrows), which have matching contoured facets along the primary distal fibular epiphysis (curved blue arrows). 3D_WATSc: 3D water selective cartilage scan.

To narrow the diagnosis, a left ankle MRI was performed. Initial T1 and T2 weighted MRI with and without contrast revealed the presence of chronic-appearing well-defined ossific fragments of the distal fibular epiphysis without surrounding edema or inflammation. 3D_WATSc MRI revealed fragments that were surrounded by non-articular cartilage. In addition, the distal fibular epiphysis demonstrated facets corresponding to each fragment in a non-linear morphology, suggesting a developmental variant rather than an old fracture (Figure 3). The left ankle MRI also demonstrated that pain was not secondary to movement at the synchondrosis between the lateral malleolus and the os subfibulare as there was no edema in this area. The MRI confirmed that symptoms were due to impingement on the lateral side of the ankle with eversion. 

**Figure 4:**
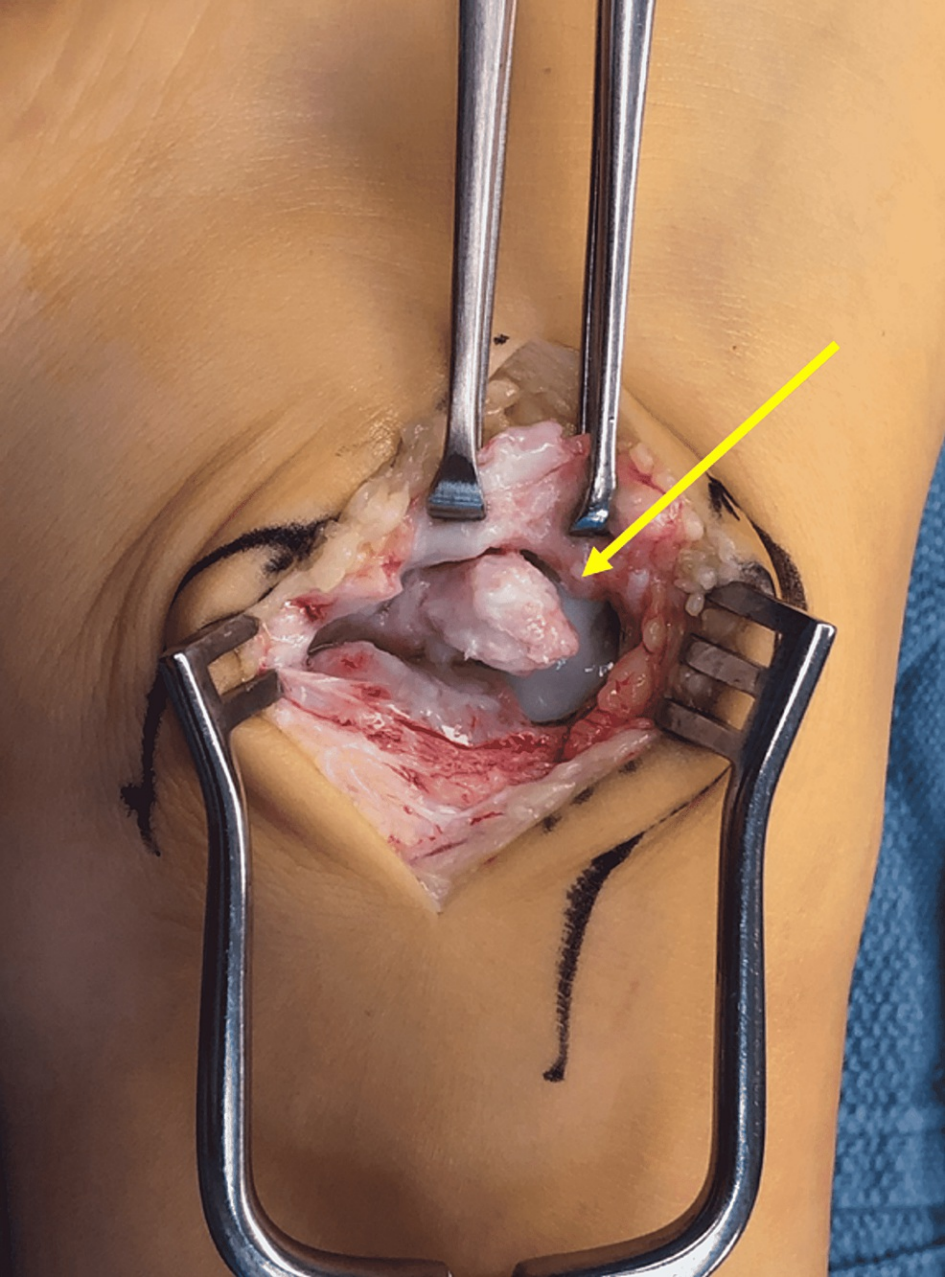
Open dissection of the left lateral malleolus and subfibulare area with the arrow (yellow arrow) pointing to the os subfibulare.

**Figure 5:**
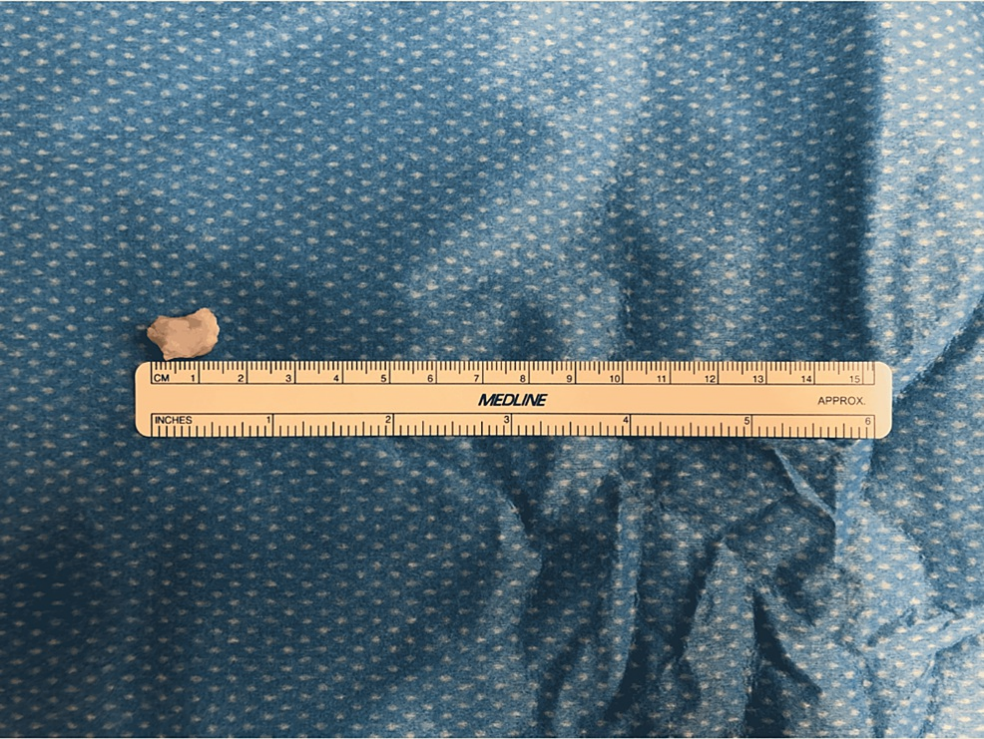
Excised accessory ossicle measuring 1.5 × 2 cm in size.

Non-operative management with physical therapy was completed to improve ankle strength and reduce lateral-sided ankle pain. Given the poor response to conservative treatment, we reached the decision to perform surgery. During the operation, open dissection was performed using a lateral approach to the left lateral malleolus. A 1.5 × 2 cm ossicle in the subfibulare area with three fragments (Figures 4, 5) was excised. The lateral ligaments were reflected inferiorly of the lateral malleolus to access the large os subfibulare. A modified Broström procedure was performed to reconstruct the lateral ligaments anatomically after ossicle excision. Postoperatively, the patient was placed in a short-leg weight-bearing cast with crutch assistance.

The patient recovered with no complications and reported resolution of ankle pain six weeks after the operation. She was then transitioned to a CAM boot and completed physical therapy with the aim of improving left-sided ankle strength, range of motion, proprioception and gait. Ten months after the operation, the patient was still free of pain with no symptoms of lateral ankle instability with only mild swelling after prolonged activity. Radiographs taken after ten months showed no osseous abnormalities. We will continue to follow up on her progress.

## Discussion

Os subfibulare can be especially debilitating for children and adolescents when symptomatic [4-7]. The lateral ankle pain and instability from os subfibulare can hinder play and complicate daily activities [8]. Therefore, prompt diagnosis and treatment should be initiated in symptomatic patients with os subfibulare. While previous studies have stressed the role of various imaging modalities including sonography, radiography, and MRI in the investigation of ankle pathologies (Table 1), we believe that the inclusion of WATSc MRI sequences provides a distinct picture of the os subfibulare, which informed our approach to treatment.

**Table 1:** Advantages and disadvantages of various imaging modalities as it relates to the investigation of os subfibulare.

Imaging modality	Advantages	Disadvantages
Sonography (ultrasound)	Widely available, rapid, and no risk of radiation exposure.	Unable to identify chondral lesions or chronicity of pathology and highly dependent on operator skill.
Plain radiographs	Commonly available, rapid, and provides diagnostic value.	Risk of radiation exposure and bony structure overlap can complicate diagnosis.
CT	Provides diagnostic value and inference into pathologic etiology.	Risk of radiation exposure, time-consuming, and limited availability.
MRI	Added variety of imaging sequences provides diagnostic and prognostic value and inference into pathologic etiology without risk of radiation exposure.	Expensive, time-consuming, and limited availability.

Sonography is regularly utilized for musculoskeletal assessment. The Sonographic Ottawa Foot and Ankle Rules (SOFAR) study performed by Canagasabey et al. demonstrated that ultrasound had high sensitivity and specificity in identifying significant foot and ankle fractures [9]. However, because of its limitations in identifying chondral lesions, characterization of acute versus chronic pathologies, and dependence on the operator’s skill, ultrasound is not commonly used to work up os subfibulare [9].

In comparison, radiographs are more commonly used to diagnose os subfibulare. A study by Lee et al. noted that while traumatic or congenital etiology of the accessory ossicle could not be determined from initial plain radiographs, additional images taken over time could be used to infer posttraumatic etiology based on the increasing size and roundness of the fragment [10]. However, a case reported by Lee et al. notes that radiographs can be limited by the superimposition of other bony structures at the ankle, which can obscure the diagnosis of os subfibulare [10]. A case report by Kose et al. proposed the use of computed tomography (CT) instead to more clearly visualize the os subfibulare as well as determine its etiological origin [6].

Finally, MRI techniques have also proven to be reliable not only in diagnosing os subfibulare but also in prognostication. An investigation by Kim et al. concluded that T1 and T2-weighted MRI of the ankle may be used to predict rehabilitation outcomes in patients with symptomatic os subfibulare based on the size of the ossicle, interposition of fluid signal intensity between the os subfibulare and fibula, and the presence of bone marrow edema within the ossicle [11]. Their study found that patients with large fragments of 10 mm or greater, the interposition of fluid signal intensity, and bone marrow edema were more likely to respond poorly to rehabilitation, suffer persistent symptoms, and ultimately require surgery [11]. In addition, advances in MRI technology have led to improvements in distinguishing the etiology of bony and cartilaginous pathologies [12].

To solidify our diagnosis of os subfibulare, we performed 3D_WATSc MRI sequencing to highlight the accessory ossicle at the tip of the patient’s left fibula. 3D_WATSc is an MRI sequence that uses water excitation to suppress fat resulting in greater image resolution and contrast [13]. While the fragments were somewhat well seen on standard MRI sequences, the WATSc sequence more clearly defined the fragment morphology and the surrounding cartilage aiding in the diagnosis of accessory ossicles. Specifically, the WATSc sequence allows for clear and confident demonstration of confluent cartilage signal surrounding the ossified portions of bone, which is most consistent with variant ossicle formation in a bed of confluent cartilage analog. This is particularly helpful in the pediatric population, where the distal epiphyses are incompletely ossified. While there is consideration of remote avulsion injury with bony nonunion, the characteristic low-signal fibrous union changes between the ossicles and the parent fibula were not seen in our patient. Additionally, the WATSc sequences allow for distinct characterization of the fragment morphology relative to the adjacent parent fibula. In this scenario, the three accessory ossicle fragments were distributed in the cartilaginous precursor analog with a well-organized morphology that illustrates matching contoured facets at each surface and with an overall distribution that follows the expected contour of the intact distal fibula.

We believe the use of 3D_WATSc MRI clearly defined the accessory ossicle to make the appropriate diagnosis of os subfibulare and inform treatment. In comparison to other case reports (Table 2), our case adds to the existing literature by providing an example of os subfibulare that was accurately diagnosed in an adolescent through WATSc MRI by clearly distinguishing the variant ossicle from a suspected fracture nonunion. In addition, this technique allowed us to reach the appropriate treatment for this patient. Had the WATSc images demonstrated a fracture nonunion, we would have performed an open reduction and internal fixation (ORIF) with a compression screw. However, since we were able to confirm the os subfibulare, we were able to confidently proceed with the excision. In summary, positive outcomes for patients with os subfibulare can be maximized with prompt and accurate imaging, appropriate treatment, and adequate follow-up.

**Table 2:** Comparison of os subfibulare case report findings. WATSc: water selective cartilage scan.

Authors	Title	Year of publication	Journal	No. of cases	Imaging modalities and diagnostic tools	Findings
Kono et al. [7]	Symptomatic Os Subfibulare Caused by Accessory Ossification: A Case Report	2002	Clinical Orthopedics and Related Research	1	Plain radiograph, MRI, pathological staining	A 17-year-old boy with symptomatic os subfibulare resulting from accessory ossification rather than avulsion fracture as determined by the presence of fibrocartilaginous tissue on pathology.
Vega et al. [4]	True Submalleolar Accessory Ossicles Causing Impingement of the Ankle	2010	Knee Surgery, Sports Traumatology, Arthroscopy	2	Plain radiograph, MRI, arthroscopy	A 29-year-old male soccer player and 36-year-old male soccer player with os subfibulare causing impingement of ankle soft tissue as seen through arthroscopy.
Kose et al. [6]	Intraarticular Entrapment of Os Subfibulare Following a Severe Inversion Injury of the Ankle: A Case Report	2015	Archives of Trauma Research	1	Plain radiograph, CT	A 19-year-old woman with entrapment of os subfibulare in the talotibial space resulting from an avulsion fracture as determined by CT.
Llanes et al.	Optimal Visualization of Os Subfibulare using 3D_WATSc MRI Sequencing: A Case Report	2022	Cureus	1	Plain radiograph, MRI	A 12-year-old female with os subfibulare resulting from variant ossification as determined by MRI WATSc which showed confluent cartilage signal surrounding the ossified portions of bone.

## Conclusions

Os subfibulare may be difficult to diagnose and should be explored further through appropriate imaging modalities. One of the difficulties in diagnosing and treating os subfibulare relates to the difficulty in distinguishing between a fracture nonunion and an accessory ossicle on radiographs. The utilization of alternative MRI sequences helps to more clearly define accessory ossicles and guide the patient to the most appropriate treatment. In this case, 3D_WATSc MRI clearly demonstrated this patient's os subfibulare by highlighting the confluent cartilage surrounding the ossicles, which informed the eventual decision to perform an open excision with a modified Broström procedure to treat this patient's pain and instability. Therefore, we posit that this imaging technique is useful in maximizing recovery and positive outcomes in patients with os subfibulare.
